# Parotid gland sialolithiasis: a comprehensive systematic review and meta-analysis: Title page

**DOI:** 10.1007/s00405-025-09697-y

**Published:** 2025-10-01

**Authors:** Giovanni Salzano, Veronica Scocca, Umberto Committeri, Stefania Troise, Luigi Angelo Vaira, Jerome R. Lechien, Vincenzo Abbate, Giovanni Dell’Aversana Orabona

**Affiliations:** 1 Maxillofacial Surgery Unit, Department of Neurosciences, Reproductive and Odontostomatological Sciences, University Federico II of Naples, University Federico II, Via Pansini 5, Naples, Italy; 2 Maxillofacial Surgery, Santa Maria Hospital, Unit, Terni, Italy; 3 Maxillofacial Surgery Operative Unit, Department of Medical, Surgical and Experimental Sciences, University of Sassari, Sassari, Italy; 4 Department of Surgery, University of Mons, Mons, Belgium; 5 Department of Neurosciences, Reproductive and Odontostomatological Sciences, University Federico II of Naples, Napales, Italy

**Keywords:** Salivary gland, Parotid, Sialolithiasis, Endoscopic surgery, Extracorporeal shock wave lithotripsy, CT-navigation

## Abstract

**Purpose:**

Parotid sialolithiasis represents unique diagnostic and therapeutic challenges due to the anatomy of the gland and the proximity of the facial nerve. The aim of this systematic review and meta-analysis was to evaluate the efficacy and safety of various treatment modalities for parotid gland stones.

**Methods:**

A systematic review was conducted in accordance with the PRISMA guidelines. Eligible studies were identified by searching PubMed/MEDLINE, the Cochrane Library, Scopus and Google Scholar. Eligible observational studies and clinical trials reporting on patients with parotid stones were included. The outcomes assessed included stone-free rate, symptom improvement and the presence of any complications. A single-arm random-effects meta-analysis was performed, focusing on endoscopy-only, endoscopy-assisted and extracorporeal shock wave lithotripsy (ESWL). Bias risk was assessed using the Newcastle–Ottawa Scale.

**Results:**

A total of 42 studies involving 1,559 patients were analyzed. Endoscopy-assisted removal showed the highest stone-free rate (93%, 95%CI: 90–96) and symptom improvement (91%, 95%CI: 92–99) (*p* < 0.05). Combined endoscopic-external approaches were effective for complex stones but had higher complication rates (24%, 95% CI: 14–37). ESWL had a lower stone-free rate (58%) but aided long-term symptom control (*p* < 0.05). CT-navigation did not significantly enhance clearance (80%). Most complications were minor; no permanent facial nerve injuries were reported. The quality of the evidence was limited by heterogeneity and the lack of any randomized trials.

**Conclusions:**

Endoscopy-assisted and combined approaches offer effective, gland-preserving options for parotid sialolithiasis. ESWL and laser techniques remain adjunctive but warrant further research. Prospective, standardized trials are needed to define any optimal management strategies.

## Introduction

Salivary duct obstruction is a common disorder, most frequently attributed to sialolithiasis, ductal stenosis and mucous plugs [1]. Sialoliths alone are responsible for 60–70% of cases [2]. While the submandibular gland is more commonly affected, approximately 20% of symptomatic stones are located in the Stenson’s duct or its branches, making parotid gland involvement a relevant consideration in clinical practice [3].

The primary aim of sialolithiasis treatment is stone removal while preserving glandular function. Over recent decades, the management of parotid sialolithiasis has evolved significantly, driven by advances in diagnostic imaging and the development of minimally invasive techniques. Historically, the treatment often involved invasive surgical procedures, including parotidectomy, particularly in cases of deeply located or intraparenchymal stones [4]. While effective in stone removal, these approaches carried significant risks, such as facial nerve injury, visible scarring and a prolonged recovery [5, 6]. The associated morbidity highlighted the need for a paradigm shift toward gland-preserving, conservative strategies that maintain salivary gland function while minimizing complications. Key to this transformation has been the introduction of sialendoscopy, which allows for a direct visualization and removal of ductal stones through natural orifices, often without the need for external incisions [2]. Combined with imaging modalities such as high-resolution ultrasound, these minimally invasive techniques now play a central role in the management of parotid stones.

The treatment for symptomatic parotid lithiasis depends on several factors, including the stone size, location and impaction and the available technical resources. Pure sialendoscopy achieves high success rates (76–86%) for both submandibular and parotid stones [7]. However, it may fail in cases with large stones or proximal duct involvement, especially when distal strictures or stenosis are present [8]. Similarly, intra- and extracorporeal shockwave lithotripsy are generally effective for stones smaller than 7 mm, but show a failure rate of approximately 10%, primarily in cases involving larger or impacted stones [9]. In such cases, sialendoscopy-assisted approaches—transoral or transfacial—may be necessary to ensure complete stone removal while preserving gland function. These techniques preserve the parotid gland by combining endoscopic precision with external surgical access, offering an effective solution for difficult cases while minimizing the risks of more invasive procedures [8]. Efforts to further enhance surgical precision have led to the exploration of intraoperative guidance systems. Ultrasound has been explored to assist in locating difficult stones, but its effectiveness is limited by factors such as indirect visualization and operator dependency [10]. In contrast, CT-based surgical navigation—widely used in anterior skull base and sinus surgery for its high precision—has recently been adapted to support combined approaches for complex salivary stone extractions, aiming to enhance accuracy and improve outcomes [11].

The aim of this systematic review and meta-analysis was to evaluate the effectiveness, safety and outcomes of current surgical strategies for the management of parotid sialolithiasis. It will provide a comprehensive overview of current treatment strategies for parotid sialolithiasis.

## Materials and methods

This study was conducted in accordance with the Preferred Reporting Items for Systematic Reviews and Meta-Analyses (PRISMA) guidelines. Since it involved a review of previously published studies, neither ethics approval nor informed consent were required. Additionally, the review was registered in the PROSPERO database under the ID number CRD420251068624.

### Search strategy

The study search covered the years 1950–2025 and included PubMed/MEDLINE, the Cochrane Library, Scopus, Embase and Google Scholar. The search was conducted independently by two investigators (V.S. and G.S.). Relevant keywords, phrases and MeSH terms were tailored to meet the specific requirements of each individual database. The search strategy used was “(parotid stone OR parotid lithiasis) AND (sialoendoscopy OR combined approach OR transcutaneous OR lithotripsy)”. Next, a cross-reference search of the selected articles was conducted using the snowballing method to ensure the retrieval of all possible studies. The electronic database search was conducted from 22nd May 2025 to 28th May 2025.

### Eligibility criteria

This systematic review and meta-analysis was carried out in accordance with PICOS. Studies with mixed cohorts (i.e., including lithiasis in other major salivary glands) were included only if subgroup data on the target population were available.

### Inclusion criteria

#### Patients (P)

Patients with parotid sialolithiasis (no age or (sub-)type of disease restriction).

#### Intervention (I)

- pure endoscopy,
- intraoral or transfacial endoscopy-assisted,
  - CT navigation-assisted.
- endoscopic laser treatment YAG-holmium laser,
- extracorporeal shockwave lithotripsy.

#### Comparison (C)

Between treatments modalities. All the studies that met the intervention and population criteria were included, regardless of whether a comparison between treatment modalities was made within the same study.

#### Outcomes (O)

- Primary outcomes: success rate defined as symptom-free (i.e., the number of symptom-free patients divided by the total number of treated patients) and stone-free (i.e., the number of stone-free patients divided by the total number of treated patients).
- Secondary outcomes: complication rate (i.e., the number of patients who experienced treatment-related adverse events out of the total number of treated patients), location and size of stones, incidence of sialoadenectomy.

#### Study design (S)

Retrospective and prospective cohort studies, case–control and cross-sectional.

studies and RCTs.

### Exclusion criteria

Studies were excluded if were not available in full-text form, to ensure access to complete methodology, data and results; included fewer than 5 patients, to minimize the risk of bias and ensure the robustness of the analysis; they were not in English, to avoid translation-related bias; if they were review articles, case reports, conference abstracts, letters to the editor and book chapters.

Once the selection criteria had been defined, 2,114 articles were screened, with 42 articles meeting the inclusion criteria.

### Data collection process

References from the identified databases were merged and duplicates were removed using the reference management software EndNote^®^ 21 (version 21.5). The articles were screened for relevance based on title and abstract, with those deemed appropriately selected for full-text review. Any disagreements between the screening authors were resolved through discussion until a consensus was achieved. Systematic data extraction from the included studies was performed using a structured form, with the data archived in a customized Excel^®^ (Microsoft Corp, Seattle, WA, USA) spreadsheet. One author (V.S.) independently compiled a standardized form to extract the following characteristics from the included studies: authors, year of publication, country, study design, number of patients, mean age, mean follow-up time, treatment strategy, mean stone size, stone location, number of stones removed, number of patients with symptom improvement, complications and parotidectomies. The accuracy of the extracted data was verified by a second author (G.S.).

### Data synthesis and analysis

All the articles included in the qualitative analysis were then included in the meta-analysis.

All clinical measures were reported as provided by the individual studies. When the mean follow-up time was not available, the median measure was used.

A single arm meta-analysis was performed for the stone free rate, symptom improvement rate, complication rate and mean stone size according to the technique of choice. The results were presented as pooled estimates with 95% CIs, and a forest plot was generated for each outcome. To stabilize any variance in the analysis of proportions, the Freeman–Tukey double arcsine transformation was applied.

The Cochran’s Q test was applied to assess the degree of heterogeneity between the studies and I^2^ was calculated as a measure of heterogeneity. The I^2^ value represents the percentage of total variation between the studies caused by heterogeneity rather than by chance. According to the Cochrane criteria, values from 0 to 40% may represent low heterogeneity, 30–60% moderate heterogeneity, 50–90% substantial heterogeneity and 75–100% considerable heterogeneity.

A random-effects model was used for all meta-analyses, if the true effect size may vary across the studies due to differences in the study populations, methodologies or other sources of variability. This model accounts for both within-study and between-study heterogeneity, providing more conservative and generalizable effect estimates.

All the analyses were performed using the R software for statistical computing (R version 4.4.2; “meta” and “dmeta” packages). Any differences in categorical outcomes (e.g., stone-free rate, symptom improvement, complication rates) between the treatment groups were assessed using the chi-square test.

### Risk of bias assessment

Two authors (V.S. and G.S.) assessed the quality of each study using the Newcastle–Ottawa Quality Assessment Scale, since all the included studies were observational cohort or case–control studies. A sensitivity analysis was conducted in this review when more than four studies were available for a given outcome, in order to assess the robustness of the pooled estimates and to explore the impact of potential sources of heterogeneity. Publication bias was assessed using funnel plots and Egger’s linear regression test when at least 10 studies were available, in accordance with the Cochrane guidelines.

## Results

### Study selection

**Fig. 1:**
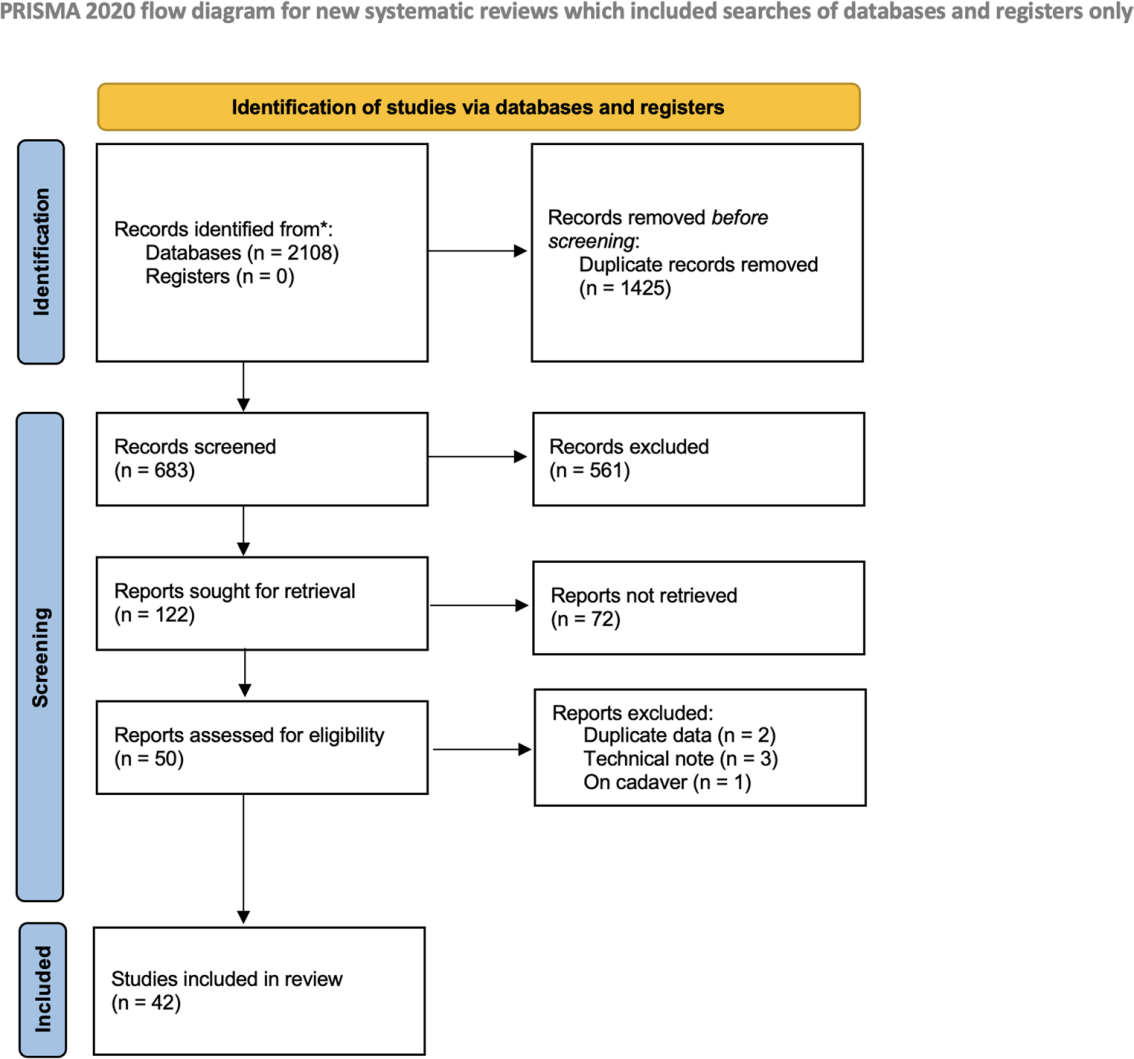
PRISMA flow diagram

The study selection process is summarized in Fig. 1. Following our comprehensive search and the exclusion of duplicate studies, 683 articles were identified. A total of 122 articles remained after screening by title. After screening by abstract content, 50 articles were read in full. Two articles were excluded because they were book chapters [12, 13]; two articles [14, 15] were excluded due to duplicate data; three articles were excluded as they were technical notes [11, 16, 17]; and one article was excluded because it was conducted on cadavers [18]. Therefore, a total of 42 publications were included in the qualitative and quantitative (meta-analysis) synthesis.

### Description of the studies

The general characteristics of the studies are shown in Table 1. Twenty-one studies were retrospective (*n* = 21/32, 65.6%), while eleven were prospective (*n* = 11/32, 34.4%). One study was published in the 1990 s, seven in the 2000 s, twenty-one in the 2010 s and twelve in the 2020s.

**Table 1:** Summary of included studies. * = Median

First Author, Year	Country	Study Design	No. (Male)	Mean Age (range)	Mean Follow-up (range)	Technique	Mean stone size (range)	Stone location	Complications	Parotidectomies
Almeida-Parra F et al., 2025	Spain	Retrospective	10 (2)	41.8 (19–67)	17mo	purely endoscopically	7.4 mm (3.5–6.5)	6: hilum, 4: duct	1: stenosis	2
Gaffuri M et al., 2025	Italy	N/A	22 (19)	53 (32–73)	18mo (1–59)	CT navigation-assisted transfacial removal	7.4 mm (4–14)	All within the gland parenchyma in a secondary ductal branch	3: sialocele	6
Hafrén L et al., 2024	Finland	Retrospective	49 (25)	55.2 (30–88)	30.5mo (0-156)	purely endoscopically, intraorally with the aid of sialendoscopy, or transfacially endoscopy-assisted	6.9 mm (3–15)	17: intraglandular 4: hilar 28: ductal	4: stenosis	0
Nguyen HL et al., 2024	Vietnam	Prospective	21 (13)	45.2 (30–65)	3mo	endoscopic YAG-holmium laser treatment	N/A	7: main duct, 10: umble gland, 3: minor duct, 1: not detected	3: scars at the opening of the salivary gland and scars in the salivary duct 1: stenosis	N/A
Tanenbaum Z et al., 2024	USA	Retrospective	26 (19)	55.5 (40–72) vs. 56 (40–77)	106mo (68–131) vs. 107mo (81–127)	transfacially endoscopy-assisted vs. intraorally endoscopy-assisted	7.4 mm vs. 5.5 mm	1: intraglandular, 12: proximal duct, 6: middle-third duct, 6: distal duct	0 vs. 2: xerostomia 5 vs. 0: salivary fistula	1 vs. 1
Zheng DN et al., 2023	China	Retrospective	68 (48)	49 (9–77)	25mo vs. 84.5mo (3–36)*	transfacially endoscopy-assisted	6.5 mm vs. 6.8 mm (3.5–6.5)	32: middle-third duct, 23: hilum, 13: proximal-third duct	2: recurrent calculus 15: swelling 1: transient facial nerve weakness 9: salivary fistula 13: wound infection 7: wound’s dehiscence	1 (partial)
Foucque O et al., 2022	France	Retrospective	5 (4)	52.6 (24–70)	10.5mo (2–21)	CT navigation-assisted transfacial removal	11.34 mm (5–18)	4: distal third duct 1: middle-third duct	2: salivary fistula 1: parotitis	N/A
Anicin A et al., 2021	Slovenia	Prospective	6	N/A	N/A	transfacially endoscopy-assisted and CT navigation-assisted transfacial removal	6 mm (5–7)	N/A	None	0
Magdy EA et al., 2021	Egypt	Retrospective	21 (16)	40.9 (12–68)	26mo (6–62)*	transfacially endoscopy-assisted	9.1 mm (5–16)	14: proximal duct, 11: secondary parenchymal branch, 2: not detected	2: seroma 1: ductal perforation	N/A
Saga-Gutierrez C et al., 2021	Spain	Prospective	8 (6)	56.7 (40–72)	8.5mo (6–12)	transfacially endoscopy-assisted	9.6 mm (6–16)	7: anterior to the masseter line, 1: below the buccinator muscle	1: stenosis 7: swelling	N/A
Xie L et al., 2021	China	Retrospective	74 (42)	49.5 (8–85)	47.1mo (6-113)	intraorally or transfacially endoscopy-assisted	6.5 mm (2–15)	53: anterior-third duct, 35: middle-third duct, 10: posterior-third duct	2: stenosis 2: numbness of the ear	1
Singh PP et al., 2020	India	Prospective	21 (10)	36.3 (6–64)	39.7mo (28–52)	transfacially endoscopy-assisted	6.4 mm	16: proximal to masseteric bend, 4: distal-third duct, 2: hilum	2: stenosis 1: wound infection	0
Kondo N et al., 2018	Japan	Retrospective	26 (17)	(20–74)	N/A	purely endoscopically	4.5 mm (1.6–6.7)	16: front of the masseter, 5: anterolateral of the masseter, 4: posterolateral of the masseter, 6: behind of the masseter, 3: not detected	N/A	N/A
Lafont J et al., 2018	France	Retrospective	38 (13)	55.6	32.7mo	extracorporeal shockwave lithotripsy	6 mm	7: anterior-third duct, 16: middle-third duct, 17: posterior-third duct	6: parotitis 20: obstructive syndrome	N/A
Ong AA et al., 2017	USA	Retrospective	44 (17)	54 (16–82)	10.9mo	transfacially endoscopy-assisted/ultrasound skin marking	8.4 mm (2–20)	24: duct, hilum, 20: intraglandular	4: numbness of the ear 2: sialocele 2: wound’s dehiscence	N/A
Ye X et al., 2017	China	N/A	116 (51)	48 (4–90)	3.9y (0.5–10)	intraorally or transfacially endoscopy-assisted	4.4 mm	53: distal-third duct, 37: middle-third duct, 13: hilum, 13: intraglandular	3: gland atrophy 3: obstructive syndrome 1: transient facial nerve weakness 1: numbness of the ear 3: stenosis	2
Foletti JM et al., 2016	France	Retrospective	22 (15)	64.1 (3–79)	47.4mo (12–79)	intraorally endoscopy-assisted	5.5 mm	16: anterior-third duct, 8: middle-third duct, 4: posterior-third duct	2: transient facial nerve weakness 2: stenosis	N/A
Rotnágl J et al., 2016	Czech Republic	Retrospective	9 (7)	57	(12–36)mo	transfacially endoscopy-assisted	6.8 mm	N/A	2: sialocele	N/A
Samani M et al., 2016	UK	N/A	111	N/A	44mo	intraorally or transfacially endoscopy-assisted	7.3 mm (1–18)	31: masseter edge, 52: preauricular, 46: hilum	17: sialocele 3: bleeding 5: parotitis 4: transient facial nerve weakness 19: obstructive syndrome	0
Konstantinidis I et al., 2015	Greece	Prospective	12 (8)	48.8 (35–67)	N/A	transfacially endoscopy-assisted	8.1 mm (2–12)	7: proximal-third duct, 5: middle-third duct	4. numbness of the ear 7: stenosis 1: parotitis 1: mild gland hypofuntion	0
Mikolajczak S et al., 2015	Germany	N/A	10	50	15mo (6–39)	transfacially endoscopy-assisted	8.7 mm	N/A	2: sialocele	0
Zheng LY et al., 2015	China	Retrospective	29 (19)	49.55 (10–85)	2.3y (6mo-3y)	purely endoscopically or intraorally endoscopy-assisted	5.8 mm (2.1–10.7)	N/A	None	0
Capaccio P et al., 2014	Italy	N/A	8 (5)	65 (37–81)	19mo (6–45)	transfacially endoscopy-assisted	12.6 mm (8–20)	4: intraglandular, 4: duct	None	0
Desmots F et al., 2014	France	Prospective	19	43 (11–68)	N/A	extracorporeal shockwave lithotripsy	6.4 mm (3–10)	6: intraglandular, 11: ductal, 2: hilar	1: parotitis	N/A
Klein H et al., 2014	Israel	Retrospective	6	49.4 (26–90)	(4–23)mo	transfacially endoscopy-assisted	10.4 mm (5–45)	N/A	None	0
Joshi AS et al., 2014	USA	Prospective	11 (6)	47.2 (22–72)	8.9mo (6–14)	transcutaneous ultrasound-guided needle placement and open sialolithotomy.	7.6 mm (5.7–11)	10: proximal third of the duct, 1: Mid third, proximal third	1: minor ductal perforation 2: sialocele	0
Carroll WW et al., 2013	USA	Retrospective	29 vs. 14	52 (31–66)	12mo (3–26)*	purely endoscopically vs. transfacially endoscopy-assisted	4.3 mm vs. 8.7 mm	24: main duct, 5: intraglandular vs. 6: main duct, 8: intraglandular, 1: not identified	3: obstructive syndrome 2: numbness of the ear 1: salivary fistula 1: sialocele	1
Kopec T et al., 2013	Poland	Prospective	5 (0)	62 (46–73)	20.4mo (2–29)	transfacially endoscopy-assisted	N/A	2: proximal-third duct, 2: distal-third duct, 1: not detected	1: gland atrophy	0
Koch M et al., 2012	Germany	Retrospective	15 (15)	53.21 (23–69)	40.67mo (3-67.5)	transfacially endoscopy-assisted	9.2 mm (4–16)	Duct	1: stenosis 1: ductal perforation 1: sialocele	1
Zenk J et al., 2012	Germany	Retrospective	115 (91)	52.6 (18–87)	150mo (12–335)*	purely endoscopically or extracorporeal shockwave lithotripsy	7 mm (2–28)	176: distal-third duct, hilum, 30: intraglandular	N/A	8
Overton A et al., 2011	UK	N/A	55 (29)	52.5 (17.6–77.6)	3.1y (2mo to 7y)	transfacially endoscopy-assisted	N/A	N/A	23: transient numbness of the ear (13 persistent) 4: sialocele 1: usutisfied with the aesthetic result	N/A
Singh PP et al., 2011	India	Prospective	5 (3)	21	minimum 6mo	purely endoscopically	8.2 mm (7–19)	3: duct, 1: hilum, 1: not detected	None	N/A
Escudier MP et al., 2010	Italy	Prospective	64	47 (30–72)	3mo	extracorporeal shockwave lithotripsy	6.16 mm (3–11)	N/A	N/A	N/A
Karavidas et al., 2010	Israel	Retrospective	69 (34)	54 (12–82)	25.5mo (2–81)	transfacially endoscopy-assisted	7.2 mm (3–15)	47: hilum, duct	1: stenosis 1: ductal perforation 2. parotitis 1: visible scar	N/A
Schmitz S et al., 2007	Germany	Retrospective	59	N/A	35.6mo (3–83)	extracorporeal shockwave lithotripsy	5.94 mm	N/A	N/A	N/A
McGurk M et al., 2006	UK	N/A	7	50 (32–75)	10mo (6–18)	transfacially endoscopy-assisted	11 mm	N/A	7: transient numbness of the ear	0
McGurk M et al., 2005	UK	N/A	88	48 (30–72)	N/A	extracorporeal shockwave lithotripsy	6.6 mm (4–15)	N/A	N/A	1
Capaccio P et al., 2004	Italy	Consecutive patient series	88	46.7 (6–89)	57mo (6-105)*	extracorporeal shockwave lithotripsy	6.62 mm (2–36)	58: ductal, 30: hiloparenchymal	N/A	0
Escudier MP et al., 2003	UK	N/A	38	50 (42–72)	3y*	extracorporeal shockwave lithotripsy	7.1 mm (4–15)	N/A	2: parotitis	N/A
Nahlieli O et al., 2002	Israeli	N/A	12 (5)	(35–62)	(24–30)mo	transfacially endoscopy-assisted	N/A	N/A	3: atrophic gland	N/A
K ¨ulkens C et al., 2001	Germany	Retrospective	42 (21)	59 (19–67)	63mo (7–69)	extracorporeal shockwave lithotripsy	N/A	13: intraglandular, 29: duct (8: proximal, 5: middle, 16: hilum	4: swelling 9: bleeding 1: wound infection	2
Ottaviani F et al., 1997	Italy	Retrospective	24	41.8 (32–73)	N/A	extracorporeal shockwave lithotripsy	N/A	N/A	N/A	N/A

### Study results

A total of 1,559 patients with parotid sialolithiasis were included in the quantitative analysis. Among this group, 121 patients underwent interventional sialoendoscopy (*n* = 121/1559, 7.8%), 568 received extracorporeal shock wave lithotripsy (ESWL) (*n* = 568/1559, 36.4%), and 774 underwent endoscopy-assisted stone removal via intraoral or transfacial approaches (*n* = 796/1559, 51.0%). Additionally, 53 patients were treated with a combination of interventional sialoendoscopy and ESWL (*n* = 53/1559, 3.4%) [3], while 21 patients underwent endoscopic laser treatment using a holmium YAG laser (*n* = 21/1559, 1.3%) [19]. 29 of the 774 patients who underwent stone removal via intraoral or transfacial approaches were treated using a CT-navigation-assisted technique (*n* = 29/774, 3.7%).

The stone location and size were not reported in studies 14 and 2, respectively. The mean stone size was 5.88 mm (95% CI: 4.41–7.36) in the pure endoscopy group, 7.8 mm (95% CI: 7.10–8.50) in the endoscopy-assisted group and 6.29 mm (95% CI: 6.01–6.57) in the ESWL group. Reported complications included post-operative sialadenitis, persistent swelling, ductal strictures, temporary paresthesia, ductal wall perforation, salivary fistula, sialoceles, post-operative infection and bleeding. Complication data were not reported in six studies.

### Stone-free rate

**Fig. 2:**
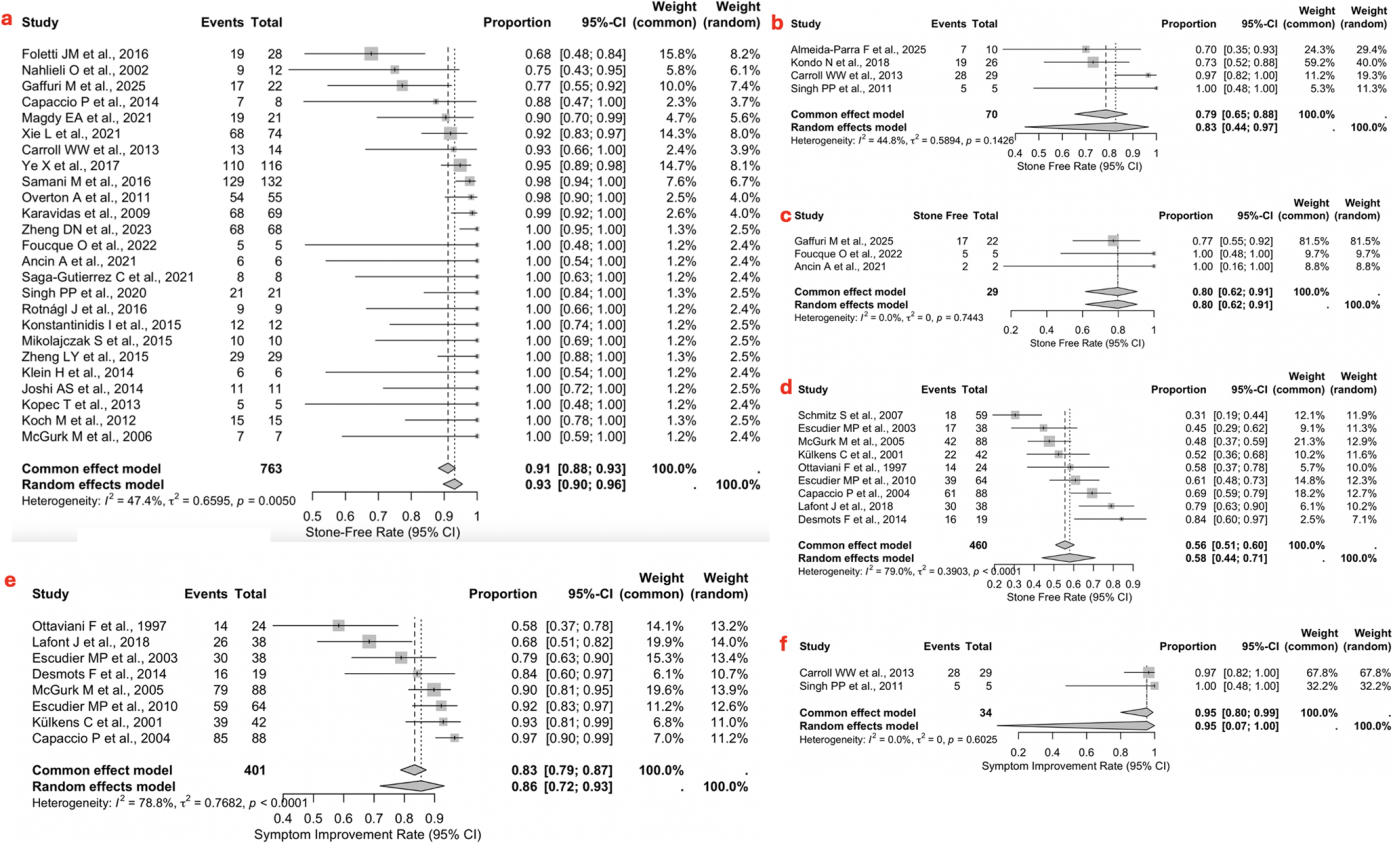
**-a**Forest plot for the stone-free rate in the pure endoscopy group;**-b** Forest plot for the stone-free rate in the intraoral or transfacial endoscopy-assisted group; **-c** Forest plot for the stone-free rate in the CT navigation-assisted group; **-d** Forest plot for the stone-free rate in the ESWL group; **-e** Forest plot for the symptom improvement rate in the ESWL group; **-f** Forest plot for the symptom improvement rate in the pure endoscopy group; **-e** Forest plot for the symptom improvement rate in the ESWL group. Abbreviation: CI, confidence interva

Regarding interventional sialoendoscopy, a pooled stone-free rate of 83% (*n* = 59/70; 95% CI: 44–97) was observed, with moderate between-study heterogeneity (I²=44.8%, Q = 0.5894, *p* = 0.1426) (Fig. 2b). In contrast, a higher pooled stone-free rate was achieved with endoscopy-assisted stone removal via intraoral or transfacial approaches, reaching 93% (*n* = 710/763; 95% CI: 0.90–0.96), and demonstrating moderate between-study heterogeneity (I²=47.4%, Q = 0.6595, *p* = 0.0050) (Fig. 2a). Three studies [20–22] reported the use of CT-navigation-assisted intra-operative localization for impacted parotid sialoliths during combined-approach extraction surgery. In this subgroup, the CT-navigation-assisted technique yielded a pooled stone-free rate of 80% (*n* = 24/29; 95% CI: 0.62–0.91), with no between-study heterogeneity (I²=0%, Q = 0.00, *p* = 0.7440) (Fig. 2c). Conversely, the use of ESWL was associated with a lower pooled stone-free rate of 58% (*n* = 267/460; 95% CI: 0.47–0.69) and considerable between-study heterogeneity (I²=81.4%, Q = 0.0232, *p* < 0.0001) (Fig. 2d). In only one study did the authors evaluate the stone-free rate following endoscopic YAG laser treatment, reporting a stone-free rate of 95% (*n* = 20/21; 95% CI: 0.86–1.00). The chi-square test demonstrated a statistically significant difference in stone-free rates between the four treatment modalities (χ²=226.73, df = 3, *p* < 0.05).

### Symptom improvement rate

**Fig. 3:**
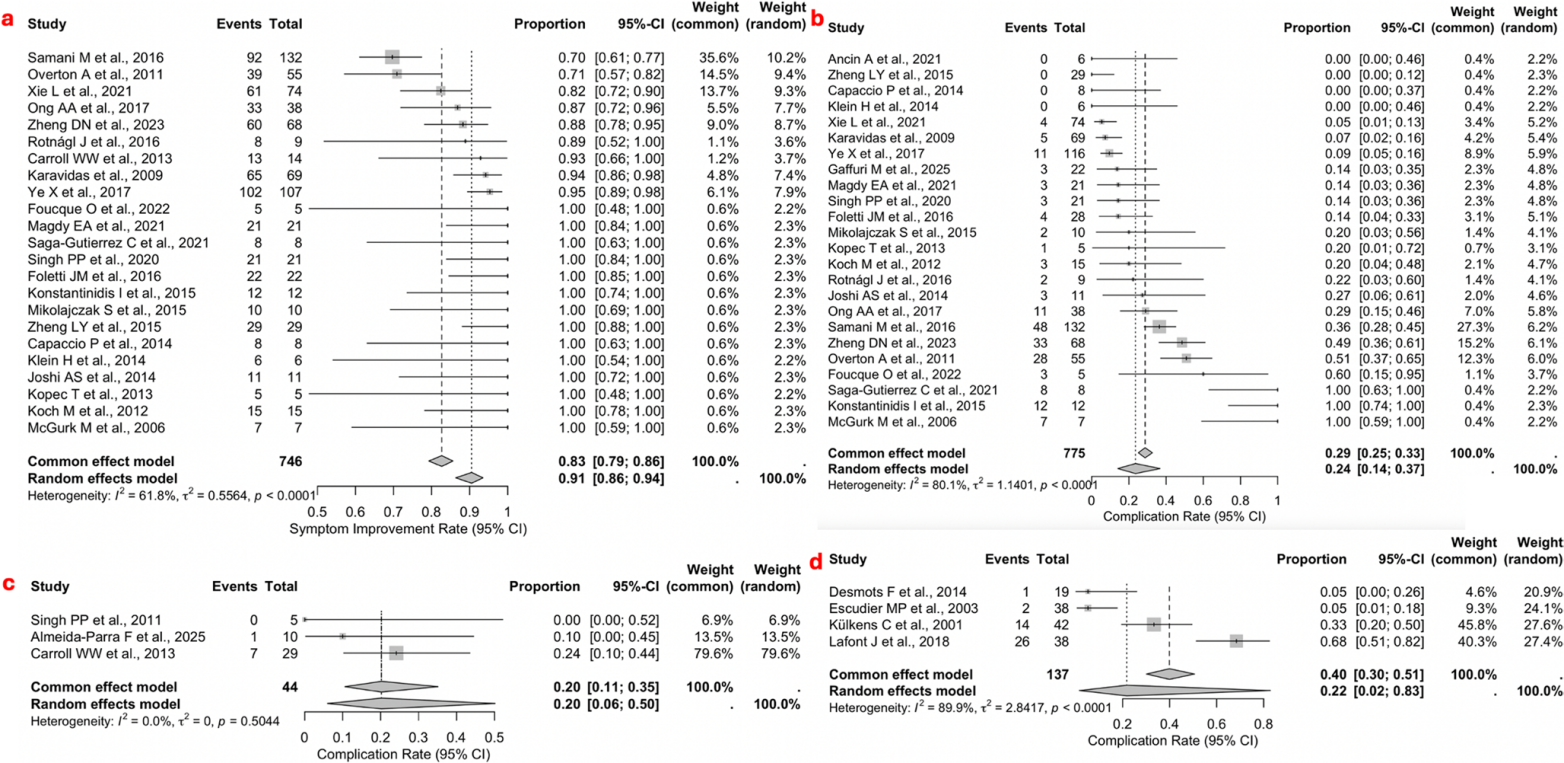
**-a** Leave-one-out sensitivity analysis for the stone-free rate in the pure endoscopy group; **-b** Leave-one-out sensitivity analysis for the complication rate in the ESWL group When at least 10 studies were available, the publication bias was assessed using funnel plot asymmetry and Egger’s linear regression test. (Fig. 5a-e)

For interventional sialoendoscopy, a pooled symptom improvement rate of 95% (n=33/34; 95% CI: 0.07–1.00) was observed, with no between-study heterogeneity (I²=0%, Q=0.00, p=0.6025) (Figure 2f). The lowest symptom improvement rate was observed in the ESWL group, with a pooled rate of 86% (n=341/401; 95% CI: 0.76–0.93) and considerable between-study heterogeneity (I²=79.9%, Q=0.0228, p<0.0001) (Figure 2e). The endoscopy-assisted group demonstrated a pooled symptom improvement rate of 91% (n=679/746; 95% CI: 0.92–0.99) with moderate between-study heterogeneity (I²=61.8%, Q=0.5564, p<0.0001) (Figure 3a). The endoscopic YAG laser treatment reported a symptom-improvement rate of 100% (n=21/21; 95% CI: 0.94–1.00). The chi-square test revealed a statistically significant difference in symptom improvement rates between the treatment modalities (χ²=14.648, df =3, p<0.05).

### Complication rate

In the endoscopy-assisted group, the pooled complication rate was 24% (n=186/775; 95% CI: 0.14–0.37), with high between-study heterogeneity (I²=80.1%, Q=1.1401, p<0.0001) (Figure 3b). The endoscopy-only group showed a pooled complication rate of 20% (n=8/44; 95% CI: 0.06–0.50), with no between-study heterogeneity (I²=0%, Q=0.00, p=0.5044) (Figure 3c). Similarly, the ESWL group had a pooled complication rate of 22% (n=43/137; 95% CI: 0.02–0.83), also with high between-study heterogeneity (I²=89.9%, Q=2.8417, p<0.0001). The chi-square test showed no statistically significant difference in complication rates between the three treatment groups (χ²=4.4876, df=2, p=0.1061).

### Risk of bias assessment

The Newcastle-Ottawa Quality Assessment Scale scores of the individual studies are shown in Table 2.

**Table 2:** Newcastle-Ottawa quality assessment scale scores of the individual studies

Study	Selection	Comparison	Outcome
Almeida-Parra F et al., 2025	xxx	xx	xxx
Gaffuri M et al., 2025	xxx	xx	xxx
Hafrén L et al., 2024	xxx	xx	xxx
Nguyen HL et al., 2024	xxx	xx	xx
Tanenbaum Z et al., 2024	xxx	x	xxx
Zheng DN et al., 2023	xxx	x	xx
Foucque O et al., 2022	xxx	xx	xxx
Anicin A et al., 2021	xxx	x	xxx
Magdy EA et al., 2021	xxx	xx	xxx
Saga-Gutierrez C et al., 2021	xxx	xx	xxx
Xie L et al., 2021	xxx	xx	xxx
Singh PP et al., 2020	xxx	xx	xxx
Kondo N et al., 2018	xxx	xx	xxx
Lafont J et al., 2018	xxx	xx	xxx
Ong AA et al., 2017	xxx	xx	xxx
Ye X et al., 2017	xxx	xx	xxx
Foletti JM et al., 2016	xxx	xx	xx
Rotnágl J et al., 2016	xx	x	xx
Samani M et al., 2016	xxx	x	xxx
Konstantinidis I et al., 2015	xxx	x	xxx
Mikolajczak S et al., 2015	xxx	xx	xxx
Zheng LY et al., 2015	xxx	xx	xxx
Capaccio P et al., 2014	xxx	xx	xxx
Desmots F et al., 2014	xxx	xx	xxx
Klein H et al., 2014	xxx	xx	xxx
Joshi AS et al., 2014	xxx	xx	xxx
Carroll WW et al., 2013	xxx	xx	xxx
Kopec T et al., 2013	xxx	xx	xx
Koch M et al., 2012	xxx	xx	xx
Zenk J et al., 2012	xxx	xx	xxx
Overton A et al., 2011	xxx	xx	xxx
Singh PP et al., 2011	xxx	x	xxx
Escudier MP et al., 2010	xxx	x	xx
Karavidas et al., 2010	xxx	x	xxx
Schmitz S et al., 2007	xx	x	xxx
McGurk M et al., 2006	xx	x	xxx
McGurk M et al., 2005	xx	x	xx
Capaccio P et al., 2004	xx	x	xx
Escudier MP et al., 2003	xx	x	xx
Nahlieli O et al., 2002	xx	x	xx
K ¨ulkens C et al., 2001	xx	x	xx
Ottaviani F et al., 1997	xx	x	xx

**Fig. 4:**
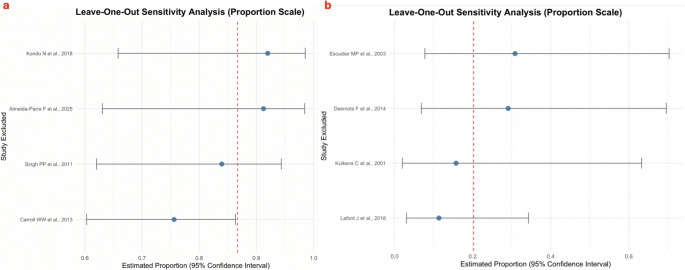
**-a** Leave-one-out sensitivity analysis for the stone-free rate in the pure endoscopy group; **-b** Leave-one-out sensitivity analysis for the complication rate in the ESWL group.

 A leave-one-out sensitivity analysis was performed when at least four studies reported an outcome to assess the influence of the individual studies. For the stone-free rate, the exclusion of any single study did not substantially alter the overall pooled estimate, which remained stable within the 95% confidence interval, indicating that the results are robust and not unduly influenced by any single study. The exception was in the pure endoscopy group, where the exclusion of Carroll WW et al., 2013 [10] resulted in a notable change in the pooled estimate, suggesting that this study has a considerable influence on the overall result (Figure4a). No substantial changes in the pooled estimates were observed for the symptom improvement rates in both the endoscopy-assisted and ESWL groups, nor for the complication rate in the endoscopy-assisted group. However, the exclusion of Escudier MP et al., 2003andLafont J et al., 2018 [23, 24] led to notable changes in the pooled complication rate for the ESWL group, indicating that these studies may significantly influence the overall estimate (Figure 4b).

**Fig. 5:**
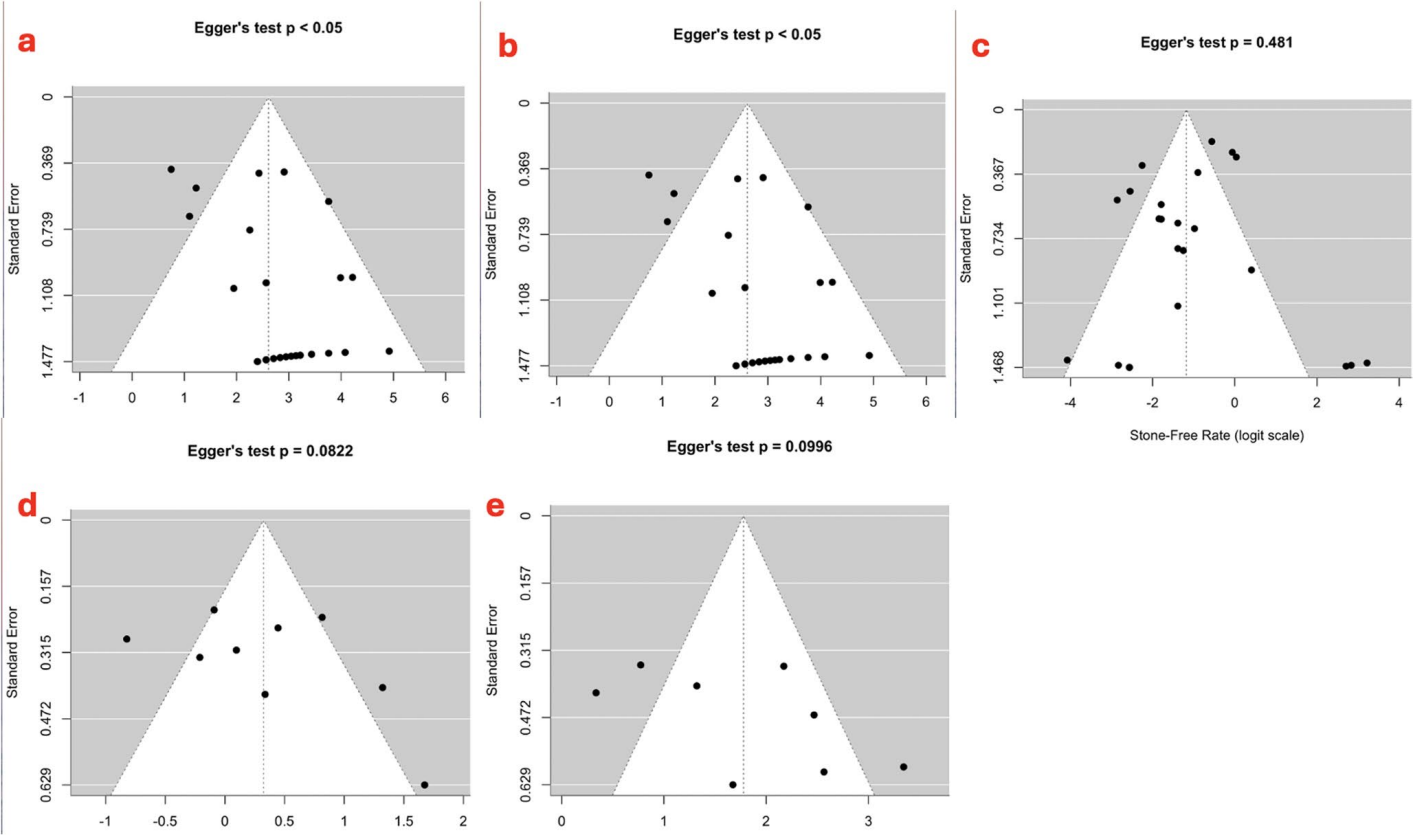
**-a**Funnel plot and Egger’s linear regression test for the stone-free rate in the intraoral and transfacial endoscopy-assisted groups; **-b** Funnel plot and Egger’s linear regression test for the symptom improvement rate in the intraoral and transfacial endoscopy-assisted groups; **-c** Funnel plot and Egger’s linear regression test for the complication rate in the intraoral and transfacial endoscopy-assisted groups; **-d** Funnel plot and Egger’s linear regression test for the stone-free rate in the ESWL group; **-e** Funnel plot and Egger’s linear regression test for the symptom improvement rate in the ESWL group

When at least 10 studies were available, the publication bias was assessed using funnel plot asymmetry and Egger’s linear regression test. (Figure 5a-e).

## Discussion

 This is the first systematic review and meta-analysis to comprehensively assess success and complication rates across all available treatment modalities for parotid sialolithiasis. Although less common than submandibular stones, parotid sialolithiasis presents unique challenges [1]. The complex branching pattern of the parotid ductal system, the proximity to the facial nerve and the often superficial location of the duct make both diagnosis and treatment technically demanding 12]. Furthermore, parotid stones are generally smaller, flatter and less radiopaque than their submandibular counterparts, which can complicate their detection on standard imaging and limit the effectiveness of some treatment modalities [25]. Therefore, the need to preserve gland function while ensuring complete stone removal is especially critical in the parotid region due to the risk of facial nerve damage and cosmetic concerns related to scarring. These considerations have driven the development and adoption of minimally invasive and image-guided interventions that balance efficacy with patient safety and satisfaction. 

 Among these techniques, endoscopy-assisted stone removal, using an intraoral or transfacial approach, achieved the highest success rates, with a stone-free rate of 93% and symptom improvement rate of 91%. These results underscore the effectiveness of combining endoscopic precision with targeted surgical access, especially for complex and large stones – such as those > 6 mm, intraparenchymal, adherent to the duct wall, prior procedural failures, or inaccessible due to a stenotic ostium or prior procedural failure [9, 14, 26–28].

Various external approaches have been described to address these challenging cases. Baurmarsh and Dechiara [29] were among the first to report an extra-oral parotid sialolithotomy without parotidectomy, using plain radiographs and ultrasound to localize the stone, followed by a horizontal skin incision for the extraction. Similarly, Nahlieli et al. [8]. employed endoscopic and ultrasound guidance to assist with the stone removal via a small vertical incision. These techniques are primarily applicable to large palpable stones located in the distal third of the duct. McGurk et al. [9]. described a modification of this combined approach, involving a localization of the parotid stones with a sialoendoscope and their removal through a short preauricular incision. More recent refinements include minimal incisions at the skin projection of the stone, providing precise access with reduced morbidity [15]. Another extraoral approach described in the literature is the Transoral Stensen’s Duct Approach (TSDA). According to Foletti et al.[30], TSDA is indicated for complex cases of parotid lithiasis following the failure of common minimally invasive procedures, such as sialendoscopy, but prior to considering an extraoral combined approach or parotidectomy. TSDA is particularly recommended in cases of anterior-third parotid duct lithiasis when sialendoscopy or lithotripsy has failed. However, the technique has its limitations. In cases involving more posteriorly located stones, the success rate is modest, and TSDA should generally not be indicated unless other options are unavailable.

Despite the theoretical advantages of CT-navigation-assisted approaches, our findings did not demonstrate a significant improvement in stone removal rates, with a pooled stone-free rate of 80%. Although CT guidance may enhance localization, its impact on clinical outcomes appears limited and warrants further study. Gaffuri et al. [22]. found that surgical failure was significantly associated with a stone depth greater than 12 mm (*p* < 0.05), suggesting that deeply embedded stones pose a unique challenge irrespective of the localization method. Moreover, CT-guided procedures entail increased technical complexity, higher healthcare costs and added radiation exposure—factors that must be carefully weighed when considering this approach.

 The increased efficacy of combined approaches was offset by a higher rate of complications, reflecting their more invasive nature. Notably, complication rates varied considerably between the studies, reflecting differences in patient selection, surgeon experience, complication definitions and follow-up durations. Nevertheless, no cases of permanent facial nerve injury were reported, with swelling being the most frequent complication [30, 31]. Most studies reported patient satisfaction with the aesthetic outcome [8, 32–36] Interestingly, the complication rate related to the combined approaches was similar to that of the lithotripsy group (24% vs. 22%). While ESWL demonstrated an 85% pooled symptom improvement rate, its stone-free rate (58%) was the lowest of all modalities, and repeat sessions are often required [36, 37]. Desmots et al. [36]. identified ultrasonic fragmentation of the stone as a significant predictor of treatment success (*p* = 0.021), with a positive correlation between total energy delivered and clinical cure (*p* = 0.04). While the outer layers of the stone fragment are readily broken, the mucoprotein-rich core often requires more energy and multiple sessions. The stone size, location, mobility and minor side effects were not significantly correlated with treatment success (*p* > 0.05) [36].

Laser-assisted sialendoscopy also shows promise as a minimally invasive option. One study [19] evaluated endoscopic surgery with a YAG-holmium laser for parotid sialolithiasis and reported complete symptoms resolution in 90.5% of cases, with a partial improvement in 9.5%. Post-operative ultrasound showed significant gland recovery, with 90.5% of patients satisfied after three months. The complication rate was 14.3%, mainly due to scarring at the duct orifice.

 Endoscopy-only approaches continue to play a pivotal role in both the diagnosis and conservative treatment of obstructive salivary gland disease. Their minimally invasive nature minimizes iatrogenic injury and preserves gland function, allowing for intraductal stone retrieval without the need for more invasive procedures such as papillotomy or adenectomy. Nonetheless, the learning curve is a significant barrier, with 30–50 cases typically required to achieve procedural proficiency—fewer than for many other endoscopic techniques [38]. Post-operative ductal stenosis remains the most frequent complication, with a 7.79% incidence reported by Almeida-Parra et al. [39]. Regarding the stone characteristics, stones greater than or equal to 5 mm are often amenable to endoscopic removal [28, 40], with stones smaller than 3 mm being ideal candidates. However, Kondo *et a*l [41] found no statistically significant correlation between stone size and successful endoscopic removal. Instead, stone location relative to the masseter muscle emerged as a crucial factor; stones anterior to the centre of the masseter had significantly higher removal rates with sialendoscopy alone compared to those posterior to this landmark [41].

To the best of our knowledge this study represents the most comprehensive systematic review to date concerning the treatment of parotid sialolithiasis. Its main strength lies in the transparent and rigorous methodology adopted, based on a pre-specified and registered protocol in accordance with the Cochrane Handbook and the PRISMA 2020 statement. A thorough and sensitive literature search was performed and study selection was independently conducted by two reviewers with excellent inter-observer agreement.

An important finding of this review is the high degree of heterogeneity across studies. Variability in patient selection, stone characteristics (size, location, mobility), surgeon experience, adjunctive tools, and evolving technology likely contributed to the observed heterogeneity in pooled estimates. For example, deeply embedded or posteriorly located stones consistently showed lower clearance rates regardless of technique, and complication rates varied with surgeon expertise and definitions used. Such heterogeneity limits the precision and generalizability of the pooled outcomes, and underscores the need for standardized reporting of both success and complications in future studies.

Another key limitation is the quality of available evidence. All included studies were observational; no RCTs were identified. Small sample sizes, retrospective designs, and heterogeneous outcome definitions reduce the strength of recommendations that can be drawn. The absence of RCTs means treatment strategies remain largely guided by expert opinion, single-center experience, and technological availability rather than high-level evidence.

Future research should focus on well-designed prospective studies with standardized definitions of success, complications, and follow-up. Multicenter collaboration could help overcome small sample sizes and allow stratification by stone characteristics. Importantly, RCTs comparing endoscopy alone, ESWL, and combined approaches are needed to define the optimal treatment algorithms. Transparent reporting of conflicts of interest and technology-related bias will also be essential.

Despite these limitations, the present systematic review and meta-analysis provides a valuable synthesis of the current evidence regarding the various treatment options for parotid sialolithiasis.

## Conclusions

This systematic review and meta-analysis offers the most comprehensive evaluation to date of treatment modalities for parotid sialolithiasis, highlighting the evolving landscape of minimally invasive and gland-preserving techniques. Endoscopy-assisted approaches have demonstrated the highest success rates, especially for large or complex stones, while endoscopy-only methods remain valuable for smaller, more accessible stones. ESWL remains a non-invasive alternative, though with lower stone-free rates and a higher need for repeat interventions. Emerging technologies such as laser-assisted sialendoscopy and image-guided surgery show promising results but require further validation. Future well-designed prospective studies with standardized outcome reporting are essential to refine the treatment algorithms and guide evidence-based clinical decision-making. Until then, the integration of sialendoscopy with adjunctive or combined approaches remains the cornerstone of the modern, function-preserving management of parotid sialolithiasis.
